# Antibacterial Activity and Mechanism of Action of Motuo Citron Essential Oil on *Staphylococcus aureus* and *Escherichia coli*

**DOI:** 10.3390/foods15183187

**Published:** 2026-09-09

**Authors:** Keyun Pan, Feng Liu, Hao Zheng, Jianwen Zhang, Liang Li, Senbiao Shu, Zhendong Liu

**Affiliations:** 1 School of Food Science, Tibet Agricultural and Animal Husbandry University, Linzhi 860000, China; 2 Key Laboratory of In-Depth Processing and High-Value Utilization of Characteristic Agricultural and Livestock Products in Tibet Autonomous Region, Linzhi 860000, China

**Keywords:** citron essential oil, *Staphylococcus aureus*, *Escherichia coli*, antibacterial activity, metabolomics

## Abstract

This study investigated the antibacterial activity and underlying mechanisms of essential oil extracted from Motuo citron against representative foodborne pathogens, including *Staphylococcus aureus* and *Escherichia coli*. The essential oil was obtained by steam distillation, and its chemical composition was analyzed by GC–MS. Limonene, γ-terpinene, and 3-carene were the predominant monoterpenes and may contribute substantially to the antibacterial activity. The Oxford cup assay confirmed strain-specific antibacterial effects. The essential oil showed stronger activity against *S. aureus*, with an inhibition zone of 22.29 ± 0.82 mm, a minimum inhibitory concentration (MIC) of 3.75 μL/mL, and a minimum bactericidal concentration (MBC) of 7.5 μL/mL. In contrast, its activity against *E. coli* was weaker, with an inhibition zone of 11.85 ± 0.49 mm, an MIC of 7.5 μL/mL, and an MBC of 15 μL/mL. Mechanistic analyses showed that the essential oil significantly reduced bacterial ATP levels and inhibited key enzymes involved in glycolysis and the tricarboxylic acid cycle, thereby impairing energy metabolism. It also disrupted bacterial cell wall and membrane integrity, resulting in cell rupture and cytoplasmic leakage. In addition, the essential oil disturbed core metabolic pathways, including amino acid biosynthesis, lysine biosynthesis, and tryptophan metabolism, causing severe metabolic imbalance in a dose-dependent manner. Motuo citron essential oil exerts antibacterial effects through the combined disruption of cell membranes, inhibition of energy metabolism, and interference with amino acid metabolism. As a natural plant-derived antibacterial agent, it shows potential for development as a food preservative and pharmaceutical antimicrobial.

## 1. Introduction

In recent years, the overuse of antibiotics in clinical and agricultural settings has accelerated antimicrobial resistance among foodborne pathogens. In particular, multidrug-resistant strains transmitted through food pose serious threats to food safety and public health. Therefore, developing new antibacterial agents that are highly effective, low in toxicity, and less likely to induce resistance has become a global research priority [1]. According to Tibetan pharmacological theory, gastrointestinal inflammation is often associated with pathogenic bacterial invasion and mucosal irritation. Thus, inhibiting the proliferation of enteropathogenic bacteria is a key strategy for alleviating such inflammatory conditions [2]. Plant-derived antibacterial agents have considerable potential as alternatives to antibiotics because of their safety, availability, and multi-target modes of action [3,4]. Plant essential oils, which are rich in terpenes and phenolic compounds, can inhibit pathogens through multiple mechanisms and are less likely to promote resistance, making them promising natural antibacterial agents. Lu et al. systematically reviewed the potential of plant essential oils as natural antibacterial preservatives in food processing and highlighted the exploration of antibacterial essential oils from traditional medicinal plants as an important approach to controlling foodborne pathogens [5].

*Citrus medica* L. is a valuable edible and medicinal plant. Dahan et al. reviewed the diverse bioactive phytochemicals present in the *C. medica* pericarp, confirming its potential as a medicinal and edible resource [6]. Its pericarp essential oil is rich in monoterpenes, sesquiterpenes, and oxygenated derivatives [7]. The pericarp also exhibits notable preservative and antioxidant properties, and previous studies have demonstrated its antibacterial and antibiofilm activities, highlighting its potential as a source of natural antibacterial agents and bioactive metabolites [8,9]. El Sayed et al. [8] used metabolomics to identify abundant antiviral metabolites in the fruits and leaves of lowland-cultivated fingered citron (*C. medica* var. *sarcodactylis*) and *Limonia acidissima* from Egypt. Lou et al. [9] further demonstrated the antioxidant, antibacterial, and antibiofilm activities of essential oil extracted from cultivated fingered citron and its nanoemulsion formulation. Nevertheless, existing studies have focused predominantly on widely cultivated lowland *C. medica* varieties, particularly fingered citron (*C. medica* var. *sarcodactylis*), leaving several important research gaps unresolved.

Motuo citron is a unique wild endemic germplasm of *C. medica* found exclusively in the high-altitude Motuo County, Xizang Autonomous Region. Unlike artificially cultivated lowland fingered citron, this wild germplasm has undergone long-term adaptation to harsh alpine conditions, including large diurnal temperature fluctuations and intense ultraviolet radiation. These environmental stresses may substantially alter the composition and relative abundance of pericarp volatile metabolites, resulting in a distinctive chemical profile and biological activities of Motuo citron essential oil that differ from those of cultivated fingered citron. To date, no systematic evaluation of the in vitro antibacterial activity or integrated mechanistic basis of Motuo citron essential oil has been reported. This knowledge gap limits the development and high-value utilization of this distinctive Tibetan medicinal plant resource. The biological and chemical differences between wild high-altitude Motuo citron and cultivated lowland fingered citron therefore represent a key novelty of the present study.

Research on the antibacterial mechanisms of essential oils has progressed from conventional activity assays, such as inhibition-zone diameter and minimum inhibitory concentration (MIC), to multi-dimensional analyses, including cellular morphology, key enzymes of energy metabolism, and microbial metabolomics. Such integrated approaches provide a more comprehensive framework for elucidating the multi-target antibacterial effects of plant essential oils [10]. Metabolomics enables the qualitative and quantitative characterization of small-molecule metabolites and the detection of metabolic changes in response to external stress [11]. In recent years, it has been widely applied to food quality evaluation, flavor characterization, clinical disease diagnosis, and the exploration of plant-derived antibacterial mechanisms [12,13,14,15]. For example, Li et al. [16] and Wang et al. [17] used untargeted metabolomics to show that ginger and clove essential oils inhibit *Escherichia coli* and *Staphylococcus aureus*, respectively, by disrupting key bacterial metabolic pathways, providing a basis for their potential use as natural food preservatives.

Although metabolomics is a powerful tool for elucidating the molecular mechanisms underlying the antibacterial activity of plant volatiles, mechanistic studies of Citrus essential oils remain limited. Most studies focus on volatile-component identification or basic in vitro antibacterial assays, while few integrate scanning electron microscopy (SEM), energy-metabolism enzyme assays, and untargeted microbial metabolomics to systematically characterize antibacterial mechanisms across multiple biological levels. Such integrated studies are particularly lacking for essential oils derived from wild, high-altitude endemic Citrus germplasm, including Motuo citron. This dual knowledge gap, i.e., the limited antibacterial characterization of wild high-altitude *C. medica* germplasm and the lack of integrated mechanistic analyses, underscores the novelty and significance of the present study.

In this study, essential oil was extracted from *C. medica* (Motuo citron) by steam distillation. We systematically evaluated its inhibitory and bactericidal activities against two representative foodborne and gastrointestinal pathogens, *S. aureus* and *E. coli*, to scientifically validate its traditional Tibetan medicinal use for alleviating symptoms of digestive tract infections [18]. By combining SEM, quantitative measurement of energy metabolism-related key enzyme activities, and untargeted microbial metabolomics, we comprehensively elucidated the cellular and molecular mechanisms underlying the antibacterial activity of Motuo citron essential oil.

## 2. Materials and Methods

### 2.1. Materials

*C. medica* (collected in October 2024 from Motuo County, Tibet, and identified by Prof. Zheng Weilie, Tibet Agricultural and Animal Husbandry University), *E. coli* (ATCC 25922), and *S. aureus* (CMCC 26003) were provided by Shanghai Luwi Technology Co., Ltd. (Shanghai, China). ATP and commercial assay kits for phosphofructokinase (PFK), malate dehydrogenase (MDH), succinate dehydrogenase (SDH), and pyruvate kinase (PK) were purchased from SuoLaiBao Biotechnology Co., Ltd. (Beijing, China).

### 2.2. Preparation of Essential Oil from C. medica

Sample pretreatment:

The pericarp was carefully peeled without damaging the oil-rich epidermis. Fresh citron pericarp was cut into uniform pieces (1 cm × 0.5 cm × 0.2 cm). Exactly 100 g of chopped pericarp was weighed and transferred to a 1000 mL round-bottom flask. Several glass beads were added, and the mixture was thoroughly shaken to ensure homogenization. The extraction buffer was preheated and maintained at 50 °C. To facilitate the release of volatile compounds trapped within the plant cell walls, cellulase and xylanase were mixed at a 1:1 mass ratio. The total amount of the enzyme mixture was 1.5% (*w*/*w*) of the pericarp mass, and both enzymes had an activity of ≥10,000 U/g. The enzymes were dissolved in the preheated buffer, and the pH was adjusted to 4.8. The pericarp suspension was enzymatically hydrolyzed at 50 °C for 1 h. After enzymatic treatment, steam distillation was continuously performed for 3 h to extract the volatile essential oil.

Separation and storage:

The oil–water mixture collected in the distillation receiver was transferred to a separating funnel and allowed to stand for 2 h for complete phase separation. The lower aqueous phase was slowly drained until the concave meniscus of the upper essential oil layer reached the zero-graduation mark of the funnel. The separated essential oil was transferred to centrifuge tubes, weighed, and recorded immediately. Purified essential oil samples were stored at −80 °C in an ultra-low-temperature freezer until further analysis. The extraction yield of Motuo citron essential oil was calculated as follows:

Essential oil yield (%) = (mass of obtained essential oil/initial mass of citron pericarp) × 100%.

### 2.3. Analysis of Physical and Chemical Indicators of Citron Essential Oil

The refractive index was determined at 20 °C using an Abbe refractometer (Model TD-92, Zhejiang Top Instrument Co., Ltd., Hangzhou, China), and relative density was measured using a digital densitometer. All measurements were performed in triplicate. The relevant Chinese national standards, GB/T 14454.4-2008 [19] and GB/T 11540-2008 [20], are listed in the References rather than in the experimental text.

### 2.4. GC-MS Identification of Citron Essential Oil Ingredients

GC conditions:

Column: Agilent DB-5ms capillary column (30 m × 0.25 mm × 0.25 μm); carrier gas: high-purity helium at a constant flow rate of 1.0 mL/min; injector temperature: 280 °C; split ratio: 30:1; injection volume: 1 μL. The essential oil samples were diluted with chromatographic-grade n-hexane before injection.

Oven program: initial temperature, 40 °C (hold for 2 min); ramp at 10 °C/min to 300 °C (hold for 2 min).

MS conditions:

Interface temperature: 280 °C; ion source temperature: 250 °C; quadrupole temperature: 150 °C. Ionization mode: EI at 70 eV; full-scan range: *m*/*z* 20–500.

Compound identification:

Compound identification was based on mass spectral matching, Kovats retention index comparison, and verification against authentic standards. Mass spectra were matched against the NIST 17 standard spectral library, with a minimum similarity threshold of 85% [21].

### 2.5. Antibacterial Activity of Essential Oil

#### 2.5.1. Oxford Cup Agar Diffusion Assay

*S. aureus* and *E. coli* standard strains stored at −80 °C were revived as follows. A small amount of frozen strain suspension was streaked onto LB agar plates and incubated at 37 °C for 16 h. Single colonies were then picked and cultured in liquid LB medium at 37 °C with shaking at 180 rpm for 12 h to prepare the working bacterial suspensions before each antibacterial test.

Following the previous methods of Han et al. [22] and Esteki et al. [23], with modifications, nutrient agar was poured into plates, evenly spread, and allowed to solidify completely. Oxford cups were then placed evenly on the agar surface. A total of 100 μL of essential oil solution, dissolved in 1% Tween 80, was added to each Oxford cup. An equal volume of sterile 1% Tween 80 was added to a separate Oxford cup on each plate as a solvent negative control to exclude potential antibacterial effects of the solubilizer. A 100-μL loading volume was used because the viscous essential oil diffuses slowly through the dense pericarp-derived agar matrix; this volume ensured sufficient diffusion of volatile oil components to produce measurable inhibition zones. After incubation at 37 °C for 24–48 h, inhibition-zone diameters were measured using a digital Vernier caliper (Model 0–150 mm, Guilin Guanglu Digital Measurement and Control Co., Ltd., Guilin, China).

#### 2.5.2. Determination of MIC and MBC

Following the methods of Owuama [24] and Aamer [25], with modifications based on the CLSI M07-A10 guideline, the broth two-fold dilution method was used to determine the MIC and minimum bactericidal concentration (MBC). The essential oil was dissolved in sterile 1% Tween 80 and serially diluted in LB broth to obtain final concentrations of 60, 30, 15, 7.5, 3.75, and 1.625 μL/mL.

For each centrifuge tube, 20 μL of diluted essential oil solution, 170 μL of LB broth, and 10 μL of bacterial suspension (1 × 10^7^ CFU/mL) were mixed to a final volume of 200 μL. Two controls were included:

Growth positive control containing 10 μL of bacterial suspension and 190 μL of LB broth supplemented with chloramphenicol, which was set to verify the normal antibacterial effect of chloramphenicol and ensure the validity of the experimental system; solvent negative control containing 12 μL of sterile 1% Tween 80 and 188 μL of LB broth without bacteria or essential oil. The latter control verified that 1% Tween 80 had no bacteriostatic or bactericidal activity. A preliminary experiment confirmed that 1% Tween 80 neither promoted nor inhibited the growth of the two tested bacterial strains.

All tubes were incubated statically at 37 °C for 24 h. The lowest concentration showing no visible turbidity was defined as the MIC. For MBC determination, 10 μL of culture from each concentration was spread onto LB agar and incubated at 37 °C for an additional 24 h. The lowest concentration producing no bacterial colonies was defined as the MBC. All experiments were performed in three independent biological replicates [26]. No antibiotic positive control was included because the study focused on the intrinsic antibacterial activity of *C. medica* peel essential oil rather than quantitative comparison with commercial antibiotics. The growth and solvent controls were used to verify the reliability of the assay.

### 2.6. Enzyme Activity Assays

Following Shu et al. [27], bacterial strains were activated and cultured overnight, and the cultures were adjusted to 1 × 10^7^ CFU/mL. The cells were harvested by centrifugation, the supernatant was discarded, and the cells were resuspended in fresh medium containing essential oil at 0.5 × MIC, 1 × MIC, or 2 × MIC. The positive control contained culture medium without essential oil.

After incubation at 37 °C for 3 h, cells were centrifuged at 8000 rpm for 5 min at 4 °C. ATP levels and the activities of key enzymes (PFK, MDH, SDH, and PK) were then determined spectrophotometrically according to the kit instructions. Inhibition rate (%) = [(sample value − control value)/control value] × 100%. Each experiment was performed in triplicate.

### 2.7. Antibacterial Mechanism

#### 2.7.1. SEM

Bacterial suspensions (approximately 1 × 10^7^ CFU/mL) were prepared and incubated with essential oil at 0 × MIC (untreated control), 1 × MIC, or 2 × MIC at 37 °C for 6 h. Bacterial pellets were collected by centrifugation at 5000 rpm for 10 min at 4 °C and washed three times with sterile PBS. Cells were pre-fixed in 2.5% glutaraldehyde at 4 °C for 6 h. After three additional washes with 0.1 M PBS (pH 7.0–7.4), the samples were dehydrated sequentially in 30%, 50%, 70%, 80%, 90%, and 100% ethanol for 15 min at each concentration. Critical point drying was then performed to remove residual ethanol. The dried specimens were sputter-coated with a thin layer of gold and examined using a scanning electron microscope (SU8010, Hitachi High-Technologies Corporation, Tokyo, Japan) at an accelerating voltage of 5 kV. Micrographs were captured at magnifications of 5000× to 20,000× [28,29].

#### 2.7.2. Non-Targeted Metabolomics

Following the method of Ou et al. [30], *S. aureus* and *E. coli* were treated with citron essential oil at 1 × MIC and 2 × MIC, with untreated samples serving as controls. All bacterial samples were incubated at 37 °C with shaking at 120 rpm for 4 h, then harvested and cryopreserved for metabolite extraction.

Frozen cell pellets were thawed on ice and homogenized with pre-cooled extraction solution A, followed by vortexing and ultrasonication. After protein precipitation at −20 °C for 1 h, the samples were centrifuged at 12,000 rpm for 15 min at 4 °C. The supernatants were freeze-dried overnight, redissolved in extraction solution B, and centrifuged again. The final supernatants were transferred to LC-MS vials. Pooled quality-control (QC) samples were prepared to monitor instrumental stability.

Metabolite detection was performed using a Q Exactive Orbitrap LC-MS/MS system (Thermo Fisher Scientific, Waltham, MA, USA) equipped with an Agilent Eclipse XDB-C18 column (4.6 mm × 250 mm, 5 μm, Agilent Technologies, Inc., Santa Clara, CA, USA). The mobile phase consisted of 0.1% formic acid in water (A) and 0.1% formic acid in acetonitrile (B), with gradient elution as follows: 0–3 min, 5% B; 3–18 min, 5–95% B; 18–20 min, 95% B; and 20–21 min, 5–95% B, followed by 4 min of column re-equilibration. The flow rate was 0.3 mL/min, the column temperature was 35 °C, and the injection volume was 5 μL. MS data were acquired in full-scan mode using electrospray ionization (ESI) in both positive and negative modes.

Raw data were processed using Compound Discoverer 3.2. Metabolites were identified by matching accurate molecular masses, MS/MS fragments, and retention times against the HMDB and METLIN databases, with mass tolerances of 5 ppm for precursor ions and 0.02 Da for fragment ions.

Multivariate PCA and OPLS-DA were analyzed using SIMCA 14.1. One-way ANOVA with Tukey’s post-hoc test was performed using GraphPad Prism 9.5. Differential metabolites were screened based on variable importance in projection (VIP) > 1 and *p* < 0.05. All data were expressed as mean ± SEM.

### 2.8. Data Processing

Multivariate statistical analyses including principal component analysis (PCA) and orthogonal partial least squares discriminant analysis (OPLS-DA) were performed to analyze metabolic differences, and one-way ANOVA with Tukey’s post-hoc test was used for univariate statistical comparison. Differential metabolites were identified based on VIP > 1 and *p* < 0.05. Compound Discoverer 3.2 (Thermo Fisher Scientific, Nyingchi, China) was used for LC-MS raw data processing and metabolite annotation; SIMCA 14.1 (Umetrics, Nyingchi, China) was used for multivariate statistical analysis; GraphPad Prism 9.5 (GraphPad Software, Nyingchi, China) was used for ANOVA and multiple comparisons; Microsoft Excel 2010 (Microsoft Corporation, Nyingchi, China) was used for data calculation; SPSS Statistics 26 (IBM, Nyingchi, China) was used for statistical significance analysis; and Origin 2024d (OriginLab, Nyingchi, China) was used for data plotting and visualization. All data were presented as mean ± SEM.

## 3. Results and Analysis

### 3.1. Extraction Yield and Recovered Mass of C. medica Essential Oil

As shown in Table 1, the enzyme-assisted extraction group yielded an average of 0.48 g of essential oil per 100 g of fruit peel, corresponding to a yield of 0.48%, whereas the traditional distillation control yielded only 0.16 g (0.16%). Enzyme treatment therefore increased the essential oil yield approximately threefold, indicating that cellulase and xylanase effectively disrupted the fruit peel cell walls and promoted essential oil release. The three replicates showed minimal variation (SD = 0.01), indicating good process stability and reproducibility.

### 3.2. Physicochemical Indicators of Essential Oil

The physical properties of citron essential oil were measured at 20/20 °C. The relative density was 0.864, and the refractive index was 1.478.

### 3.3. Chemical Composition of the Essential Oil

GC–MS was used to characterize the chemical composition of citron peel essential oil obtained by steam distillation. A total of 31 volatile compounds were identified, accounting for >99% of the total chromatographic peak area. Terpenes were the predominant constituents (92.31%), followed by aldehydes (3.40%), alcohols (1.11%), esters (0.37%), and trace amounts of amines and other hydrocarbons. β-Myrcene, 3-carene, D-limonene, and γ-terpinene were the major constituents, collectively accounting for more than 90% of the total oil and defining its overall chemical profile. Although present at low concentrations, oxygenated terpenoids may contribute substantially to the characteristic aroma of citron essential oil.

The predominant monoterpene hydrocarbons are likely major contributors to the antibacterial activity of the oil. D-limonene exhibits broad inhibitory activity against foodborne pathogenic bacteria, with reported MIC_50_ values of 0.420–1.598 mg/mL [31,32]. γ-Terpinene inhibits the growth of *Salmonella enteritidis*, with MIC and MBC values of 3.125 µL/mL [33]. 3-carene exerts stronger antibacterial activity than many structurally related monoterpenes, reportedly by disrupting bacterial cell structures, binding genomic DNA, and interfering with key metabolic enzymes [34]. β-myrcene also exhibits antibacterial activity against a range of foodborne pathogens [35]. Collectively, these abundant monoterpene hydrocarbons may represent the principal active constituents underlying the antibacterial activity of citron peel essential oil.

The 31 identified volatile compounds, together with their retention times, relative contents, library matching scores, and CAS numbers, are listed in Table 2. Compounds were identified by matching their experimental mass spectra with the NIST17 standard spectral library, and the matching score for each chromatographic peak was recorded. Kovats retention indices were not determined in this study. The relative content of each volatile compound was calculated by chromatographic peak-area normalization.

### 3.4. Inhibition Zone, MIC, and MBC

**Figure 1:**
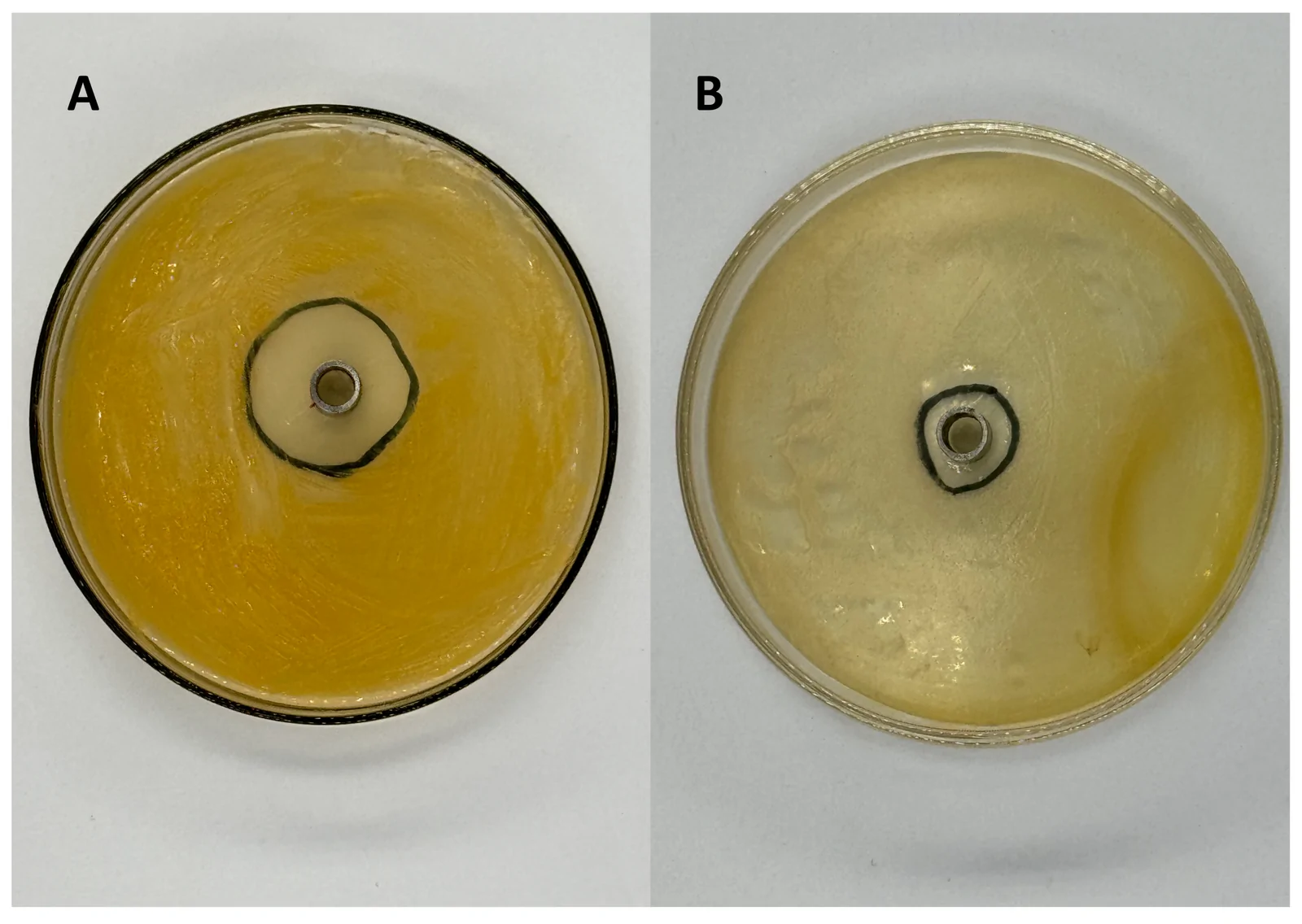
Bacteriostatic zone of citronella oil using the Oxford cup method. (**A**) *Staphylococcus aureus* (**B**) *Colon bacillus*.

Inhibition-zone diameters were measured in triplicate using a digital Vernier caliper with 0.1-mm precision, and mean values were calculated. As shown in Figure 1 and Table 3, the essential oil produced inhibition zones of 22.29 ± 0.82 mm against *S. aureus* and 11.85 ± 0.49 mm against *E. coli*. The chloramphenicol positive control produced larger inhibition zones (25.37 ± 0.63 mm and 23.23 ± 0.58 mm, respectively), confirming the validity of the assay. No inhibition was observed in the blank control, indicating no interference from the solvent or culture medium.

Consistent with the agar diffusion results, the MICs of the essential oil were 3.75 mg/mL for *S. aureus* and 7.5 mg/mL for *E. coli*, whereas the corresponding MBCs were 7.5 mg/mL and 15 mg/mL, respectively (Table 4). An MBC/MIC ratio of 2 for both strains indicated bactericidal rather than purely bacteriostatic activity. The control results, including the absence of growth in the chloramphenicol group and visible growth in the negative and blank controls, confirmed bacterial viability and the absence of experimental contamination.

Overall, citron peel essential oil exhibited clear antibacterial activity against both pathogens, with *S. aureus* (Gram-positive) being more susceptible than *E. coli* (Gram-negative). These findings prompted further investigation of the underlying antibacterial mechanism.

### 3.5. Antibacterial Mechanisms of Citron Essential Oil

#### 3.5.1. SEM Observations

**Figure 2:**
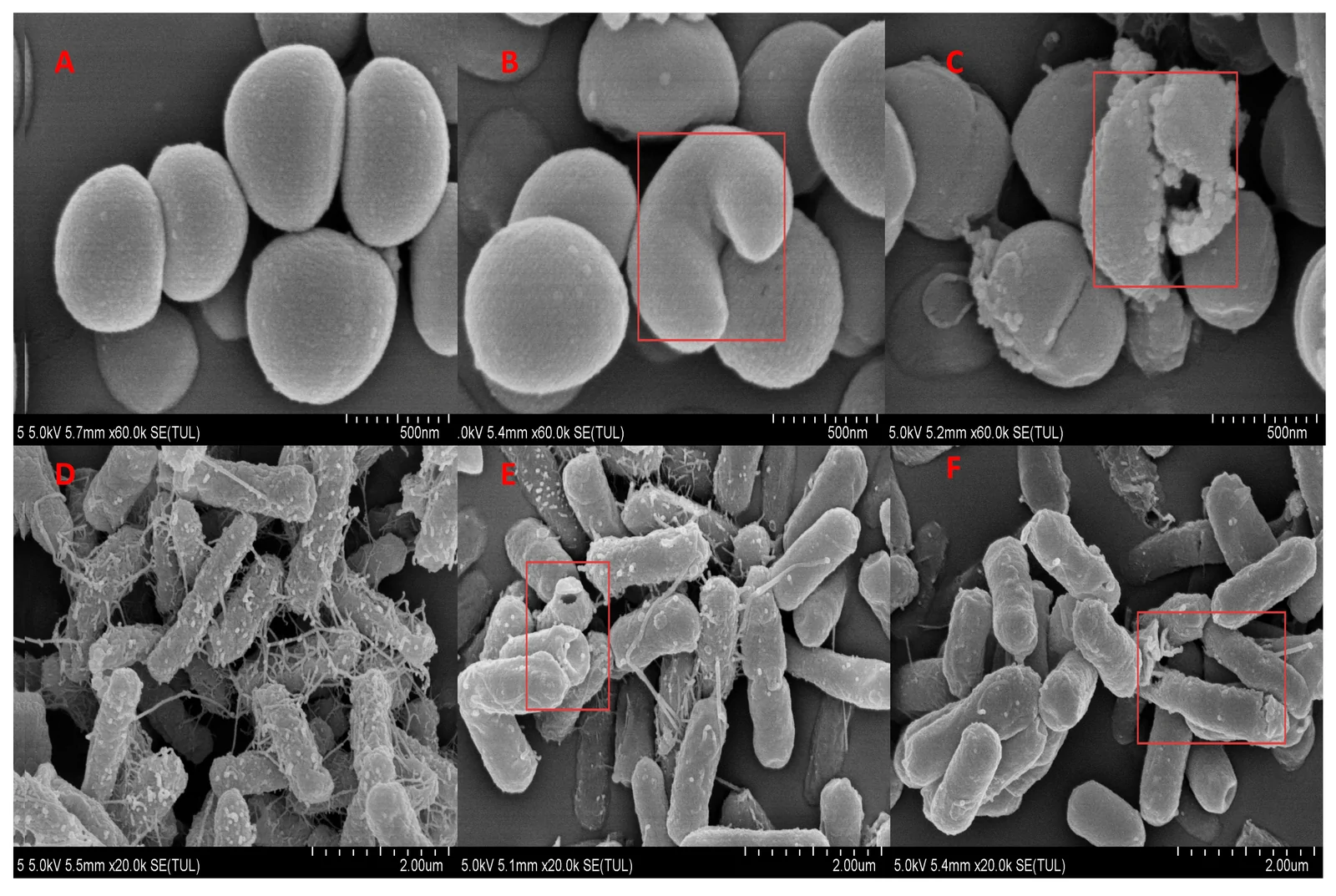
Scanning electron microscopy observation of *Staphylococcus aureus* (**A**–**C**) and *Escherichia coli* (**D**–**F**) after treatment with Citrus medica essential oil. (**A**) Untreated *S. aureus* showing intact spherical cells with smooth surface; (**B**) *S. aureus* treated with low concentration of essential oil, exhibiting deformed cell morphology (red box); (**C**) *S. aureus* treated with high concentration of essential oil, showing severe cell collapse and surface disruption (red box); (**D**) Untreated *E. coli* displaying regular rod-shaped cells with smooth surface; (**E**) *E. coli* treated with low concentration of essential oil, presenting cell indentation and partial surface damage (red box); (**F**) *E. coli* treated with high concentration of essential oil, showing cell rupture and cytoplasmic leakage (red box). Red boxes mark representative morphological damage of bacterial cells induced by essential oil.

Figure 2 shows the morphological changes in *S. aureus* and *E. coli* exposed to citron essential oil, as observed by SEM. Panels A–C show *S. aureus* treated with 0 × MIC (untreated control), 1 × MIC, and 2 × MIC essential oil, respectively, whereas panels D–F show *E. coli* treated at the same concentration gradients.

SEM observations revealed that untreated bacterial cells in Panels A and D exhibited intact, smooth, and uniform surfaces without obvious structural damage. After treatment with 1 × MIC essential oil (Panels B and E), *S. aureus* cells became wrinkled and partially ruptured, whereas *E. coli* cells showed surface depression and marked structural deformation. At 2 × MIC (Panels C and F), *E. coli* cells exhibited severe structural disruption, including fragmented residues and loss of cell integrity. Consistent with previous reports [36,37] that plant essential oils disrupt bacterial cell walls and membranes, these findings indicate that citron essential oil causes concentration-dependent membrane damage. Such damage may disrupt intracellular osmotic balance, impair metabolic enzyme activity, and ultimately lead to bacterial cell death.

#### 3.5.2. Metabolomics Analysis

##### Screening of Differential Metabolites

**Figure 3:**
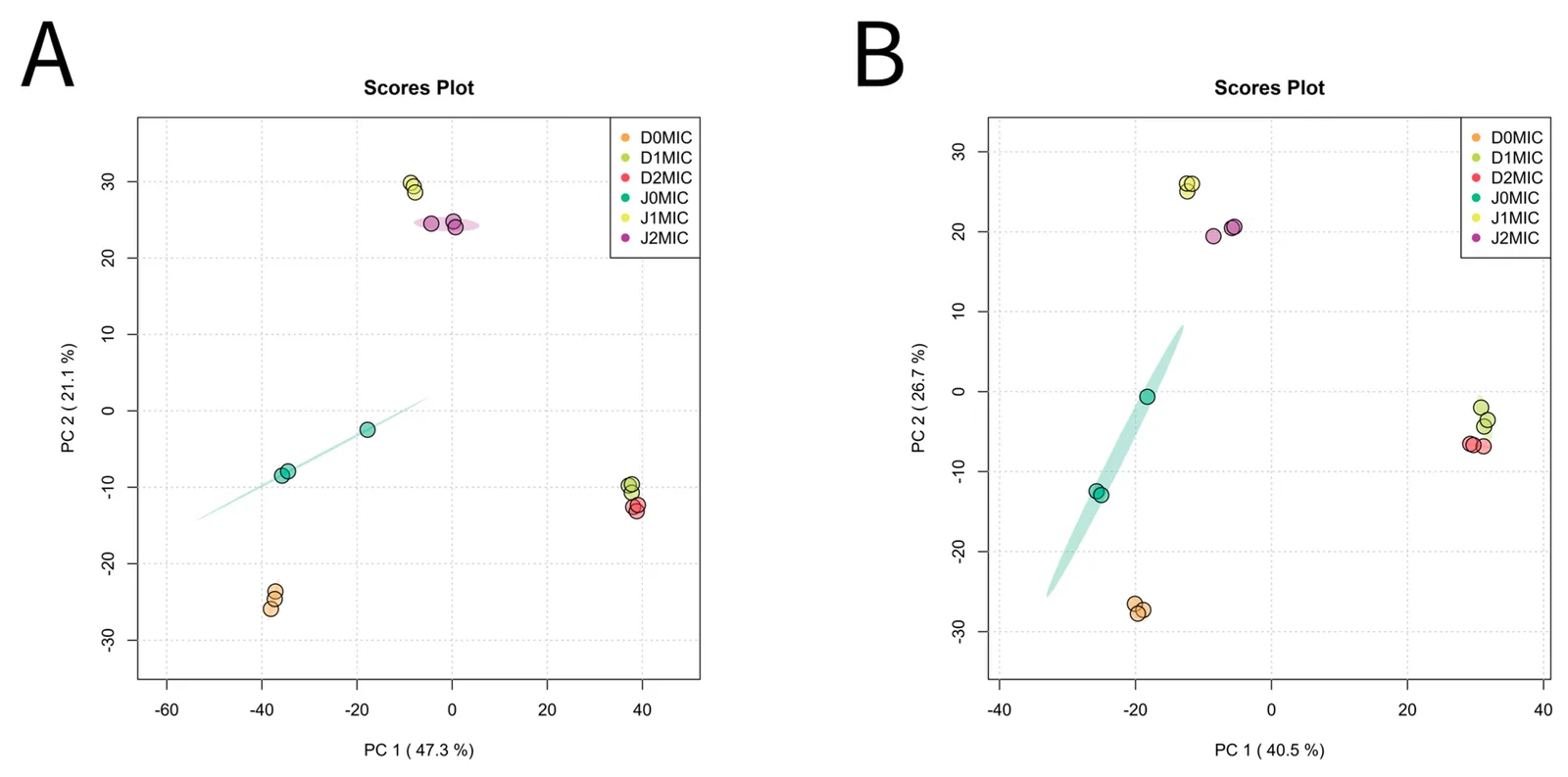
Principal component analysis (PCA) scores plot of metabolite profiles of bacterial samples treated with *Citrus medica* essential oil. (**A**) PCA-scores plot based on positive-ion mode data; PC1 = 47.3 %, PC2 = 21.1 %. (**B**) PCA-scores plot based on negative-ion mode data; PC1 = 40.5 %, PC2 = 26.7 %. Different colors represent sample groups D0MIC, D1MIC, D2MIC, J0MIC, J1MIC and J2MIC as shown in the legend.

PCA, an unsupervised multivariate statistical method, was applied to visualize inter-group metabolic discrepancies as shown in Figure 3.

Figure 3 presents the PCA score plots obtained under electrospray ionization positive mode (Panel A, where PC1 and PC2 explained 47.3% and 21.1% of the total variance, respectively) and negative mode (Panel B, where PC1 and PC2 accounted for 40.5% and 26.7% of the total variance, respectively). Under both ionization modes, biological replicates within the same treatment group clustered tightly, whereas the control and essential-oil-treated groups were clearly separated. This distinct separation indicates substantial intracellular metabolic perturbations induced by citron essential oil, consistent with previous studies by Bai et al. [38] and Yuan et al. [39], which showed that bacteriostatic agents can induce extensive metabolic disturbances in pathogenic bacteria. Notably, the first two principal components collectively explained more than 60% of the total variance in both ionization modes, confirming the robustness of the PCA models. The clear separation among groups provides a reliable basis for subsequent screening and identification of differential metabolites.

##### Changes in Differential Metabolite Expression

Volcano plots were used to visualize the statistical significance and magnitude of metabolic changes. Amino acid metabolism is an important metabolic pathway, as amino acids are essential for protein and enzyme synthesis, regulation of gene expression, and maintenance of cellular osmotic balance [40].

**Figure 4:**
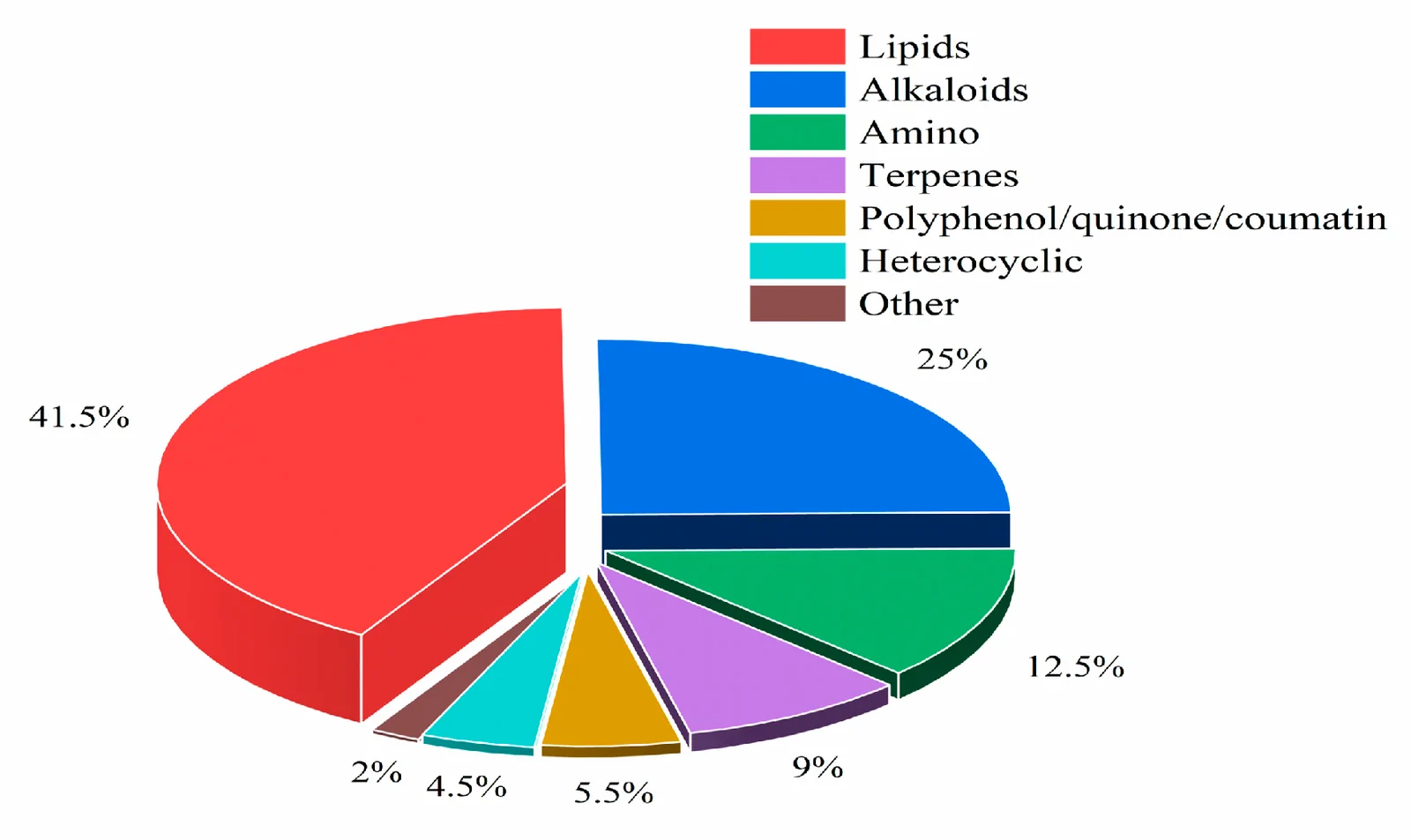
Volcano plot and heatmap of differential metabolites of citron essential oil on *S. aureus* and *E. coli*.

As shown in Figure 4, essential oil treatment markedly altered the metabolic profiles of both bacterial species. In *E. coli*, 3069 metabolites were significantly altered (1865 downregulated and 1204 upregulated) based on the criteria of FC > 2 and VIP > 1. Up-regulated metabolites were mainly lipids, including lysophosphatidylethanolamine, phosphatidylethanolamine, and diacylglycerols, whereas downregulated metabolites included alkaloids, carotenoids, exogenous compounds, and some vitamin E derivatives. In *S. aureus*, 952 metabolites were significantly altered (500 downregulated and 452 upregulated). Upregulated metabolites mainly comprised alkaloids, coumarin-related compounds, acylcarnitines, and some steroid-related compounds, whereas downregulated metabolites included lysophosphatidyl species, short peptides, vitamin-related metabolites, and some exogenous compounds. Overall, the differential metabolites in both species spanned multiple classes, including lipids, alkaloids, coumarins, peptides, and vitamins, indicating broad metabolic effects of citron essential oil.

Among the top 50 differential metabolites, lipids accounted for 41.50%, indicating that membrane lipid metabolism and fatty acid transport were the major metabolic alterations. Alkaloids accounted for 25.00%, with their levels increasing with essential oil concentration and potentially contributing to antioxidant defense. Amino acids and their derivatives accounted for 12.50% and were associated with impaired protein synthesis, whereas terpenes and polyphenols accounted for <21% and appeared to have auxiliary metabolic roles. These findings indicate that citron essential oil markedly disrupts lipid and amino acid metabolism and induces compensatory antioxidant responses involving alkaloids. The overall pattern of metabolic disturbance was consistent across treatment concentrations.

##### KEGG Pathway Analysis

**Figure 5:**
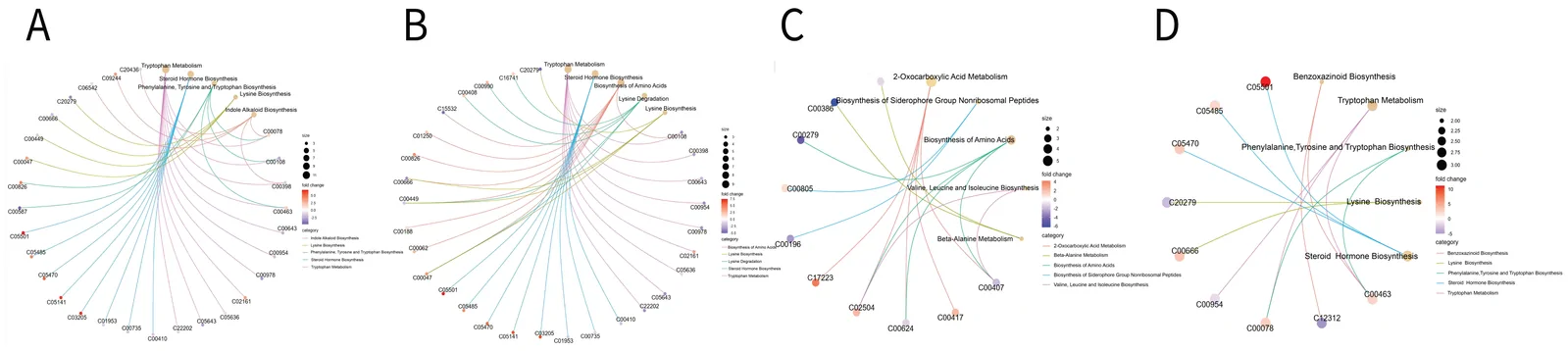
KEGG enrichment network analysis of differentially expressed metabolites after *Citrus medica* essential oil treatment. (**A**) Enrichment network of differential metabolites in the D0MIC vs. D1MIC comparison group; (**B**) Enrichment network of differential metabolites in the D0MIC vs. D2MIC comparison group; (**C**) Enrichment network of differential metabolites in the J0MIC vs. J1MIC comparison group; (**D**) Enrichment network of differential metabolites in the J0MIC vs. J2MIC comparison group. Node size represents the enrichment significance; line colors correspond to different metabolic pathways; node color gradient indicates log_2_(fold-change) of metabolites. Legends on the right of each subfigure represent the corresponding annotation information.

KEGG pathway enrichment analysis (Figure 5) was performed using the enrichment factor, *p*-value, FDR, and number of differential metabolites annotated to each pathway for quantitative assessment.

In *E. coli* treated with 1 × MIC essential oil, tryptophan and steroid biosynthesis were significantly upregulated, whereas lysine biosynthesis was markedly downregulated, as indicated by the low FDR and high enrichment factor. Cross-talk between pathways was evident through shared differential metabolites, such as C05501. Under 2 × MIC treatment, most amino acid biosynthetic pathways showed compensatory upregulation, whereas tryptophan and lysine biosynthesis were strongly inhibited; C00463 and C12312 were key differential metabolites enriched in these pathways. In *S. aureus* exposed to 1 × MIC essential oil, amino acid and energy metabolism were the most significantly enriched pathways and showed overall downregulation, with isoleucine (C00407) identified as a central hub metabolite. At 2 × MIC, benzoxazole and tryptophan metabolism were significantly activated, whereas lysine biosynthesis was inhibited, with tryptophan (C00078) and the lysine precursor C20279 acting as key regulatory nodes. Collectively, amino acid metabolism was the primary metabolic target of citron essential oil in both bacterial strains. Low-dose treatment suppressed essential metabolic pathways, whereas high-dose treatment induced secondary stress-response metabolism. Lysine and tryptophan biosynthesis were conserved stress-responsive pathways in both bacteria, and shared differential metabolites may mediate cross-talk among multiple enriched metabolic pathways.

### 3.6. Enzyme Activity

**Figure 6:**
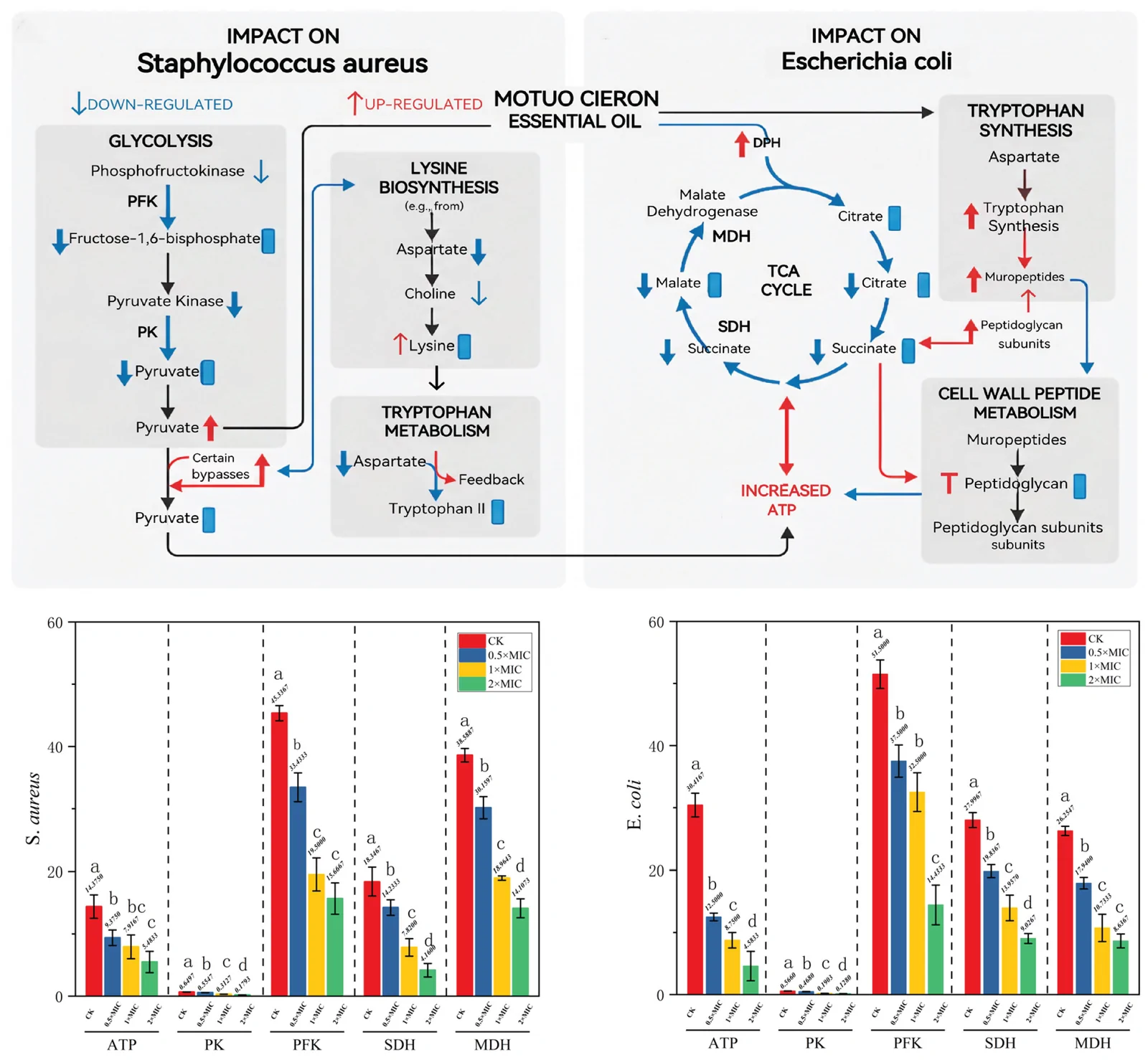
Enzymatic Activity induced by citron essential oil. a–d are the significance of their four kinds.

To further validate the inhibitory effect of citron essential oil on bacterial energy metabolism, we measured the activities of rate-limiting enzymes in glycolysis (PFK and PK) and the TCA cycle (MDH and SDH), together with intracellular ATP levels. Essential oil treatment significantly reduced all enzyme activities and ATP levels. The relative reductions, absolute values, standard deviation (SD), and *p*-values are summarized in Figure 6. ATP levels decreased by 61% in *S. aureus* and 86% in *E. coli*. PFK activity decreased by 38% and 61%, PK activity by 72% and 77%, MDH activity by 63% and 67%, and SDH activity by 77% and 67%, respectively. Figure 6 provides the complete quantitative data, including absolute activity values, SDs, and statistical significance.

These results demonstrate that citron essential oil disrupts bacterial energy metabolism through multiple targets. Inhibition of the key glycolytic enzymes PFK and PK suppresses glycolysis, thereby limiting glucose catabolism and initial energy production. Reduced MDH and SDH activities further impair the TCA cycle and oxidative phosphorylation. The combined inhibition of glycolysis and the TCA cycle ultimately restricts ATP production and causes severe intracellular energy depletion. Thus, citron essential oil exerts broad-spectrum antibacterial activity by simultaneously disrupting two central bacterial energy-metabolism pathways.

## 4. Discussion

This study confirmed that Motuo *C. medica* essential oil exhibited significant antibacterial activity against *S. aureus* and *E. coli*, with stronger inhibition against the Gram-positive bacterium than the Gram-negative bacterium. This pattern is consistent with the antibacterial activity of Rutaceae citrus essential oils reported by Medeleanu et al. [41]. Structurally, *E. coli* possesses an outer membrane rich in lipopolysaccharides (LPSs), which forms a barrier to the penetration of hydrophobic monoterpenes. In contrast, *S. aureus* has a single-layer peptidoglycan cell wall, making it more susceptible to active components such as limonene and γ-terpinene. This difference may explain the common pattern of greater sensitivity of Gram-positive than Gram-negative bacteria to essential oils.

GC–MS analysis showed that limonene, γ-terpinene, and 3-carene together accounted for more than 90% of the oil, representing the major chemical components associated with its antibacterial activity and consistent with reported composition–activity relationships of citrus peel essential oils. Compared with other citron essential oils reported in the literature, Motuo *C. medica* essential oil exhibited a distinct chemical profile, characterized by an exceptionally high limonene content (>80%) and relatively low levels of oxygenated monoterpenes such as citral and linalool. This difference may be attributed to the unique geographical and climatic conditions of the Motuo region in Tibet, as well as genetic variation among *C. medica* cultivars. Notably, although some studies have reported potent antibacterial activity in citral-rich citron essential oils, the present findings demonstrate that limonene-dominated oils also exhibit significant antibacterial activity. Terpenoids widely existing in plant resources exhibit broad-spectrum antibacterial capacity, and their functional performance can be further improved via appropriate delivery carriers [42].

Cell membrane integrity is crucial for bacterial growth and metabolism. SEM observations (Figure 2) showed that treatment with 1 × MIC and 2 × MIC caused concentration-dependent wrinkling, depressions, and irregular structural damage in both bacteria. At 2 × MIC, the characteristic filamentous morphology of *E. coli* was nearly lost, with severe membrane rupture and evidence of intracellular content leakage. These morphological changes indicate that the essential oil disrupts bacterial cell wall and membrane structures, resulting in intracellular leakage and ultimately structural collapse. Similar alterations have been reported for other citrus essential oils, including lemon and orange oils, for which membrane disruption has been identified as a major antibacterial mechanism. However, the damage observed in the present study appeared more pronounced at equivalent concentrations, potentially because the high content of monoterpene hydrocarbons in Motuo *C. medica* essential oil may promote more efficient partitioning into the lipid bilayer of bacterial membranes. Interestingly, previous studies of citron essential oils rich in oxygenated monoterpenes have also reported membrane-disruptive effects, suggesting that both hydrocarbon and oxygenated terpenes contribute to membrane damage through distinct physicochemical interactions. The present findings further support the role of limonene and γ-terpinene, the major hydrocarbon monoterpenes, in disrupting bacterial membranes by partitioning into the hydrophobic core of the lipid bilayer and increasing membrane fluidity and permeability, even in the absence of high concentrations of oxygenated compounds.

Studies suggest that bacterial death may result not only from membrane damage but also from metabolic disruption [17]. Intracellular metabolite levels can reflect the inhibition or activation of corresponding metabolic pathways. In this study, non-targeted metabolomic PCA (Figure 3) showed clear separation between the control and treated groups in both positive and negative ion modes, indicating substantial disruption of normal cellular metabolism in both bacterial species following essential oil treatment. Heatmap analysis (Figure 4) of the top differential metabolites showed that lipids were predominant (41.50%), followed by alkaloids (25.00%) and amino acids/derivatives (12.50%). These results suggest that lipid/fatty acid metabolism is a major pathway affected by essential oil stress. Amino acid biosynthesis was also significantly inhibited, whereas alkaloids may represent a compensatory secondary defense response that increases with stress concentration. Compared with metabolomic studies of other citrus essential oils, the present findings indicate a more pronounced perturbation of lipid metabolism, which may be attributable to the high proportion of monoterpene hydrocarbons that preferentially interact with membrane lipids. Previous metabolomic studies of lemon and bergamot essential oils have similarly identified lipid metabolism as a major target pathway, although the specific lipid classes affected differ, likely because of differences in essential oil composition and bacterial susceptibility.

As the primary pathway for ATP production, the TCA cycle plays a central role in bacterial energy metabolism. Enzyme assays (Figure 6) showed that the essential oil significantly inhibited the activities of the key glycolytic enzymes PFK and PK and the TCA cycle enzymes MDH and SDH, accompanied by substantial decreases in intracellular ATP levels (61% in *S. aureus* and 86% in *E. coli*). SDH catalyzes the oxidation of succinate to fumarate and transfers electrons to ubiquinone, making it essential for bacterial energy metabolism. MDH catalyzes the interconversion of malate and oxaloacetate, and its inhibition reduces NADH production, thereby impairing subsequent ATP synthesis. In this study, SDH activity decreased by 77% (*S. aureus*) and 67% (*E. coli*), whereas MDH activity decreased by 63% and 67%, respectively. These results indicate that the essential oil simultaneously suppresses key energy-metabolism steps, thereby impairing ATP synthesis. The findings are consistent with previous reports showing that tea tree essential oil inhibits TCA cycle enzymes in Penicillium species [43] and that citral disrupts TCA cycle-related enzyme activities in *Penicillium* [44]. Compared with previous studies of citron essential oils, which have primarily focused on membrane-disruptive mechanisms, the present study provides evidence that Motuo *C. medica* essential oil also exerts substantial metabolic effects by suppressing key energy-generating enzymes. This dual mechanism, involving both membrane disruption and metabolic interference, may contribute to the relatively low MIC values observed in this study compared with those reported for some citron essential oils, suggesting that its multi-target antibacterial action enhances overall efficacy.

KEGG pathway enrichment analysis (Figure 5) revealed marked perturbations in amino acid biosynthesis, particularly pathways related to lysine and tryptophan metabolism. Lysine is synthesized through the diaminopimelate (DAP) pathway, in which DAP also serves as a precursor for peptidoglycan biosynthesis and cell wall formation [45]. Consistent with the metabolomic data, lysine biosynthesis showed an overall downward trend in both strains. This reduction may impair bacterial protein synthesis and cell wall formation, potentially contributing to the severe cellular damage observed by SEM. Tryptophan is essential for protein synthesis and serves as a precursor of indole, a signaling molecule involved in bacterial biofilm formation [46]. Reduced tryptophan metabolism may therefore limit bacterial proliferation and indole production, potentially impairing biofilm maturation. Shared metabolites, including tryptophan (C00078) and the lysine precursor (C20279), act as metabolic junctions linking multiple pathways. Collectively, these metabolomic changes suggest that citron essential oil disrupts the interconnected amino acid metabolic network at multiple points, leading to broad metabolic dysfunction in pathogenic bacteria. Although previous studies of citron essential oils have mainly focused on membrane damage and oxidative stress, our metabolomic and enzymatic data indicate that amino acid metabolism is also an important target. The combined disruption of lipid, energy, and amino acid metabolism may distinguish Motuo *C. medica* essential oil from other citrus essential oils and contribute to its strong antibacterial activity.

Overall, Motuo *C. medica* essential oil exerts antibacterial effects through three complementary mechanisms: (1) disruption of cell wall and membrane integrity; (2) inhibition of key glycolytic and TCA cycle enzymes, resulting in ATP depletion; and (3) disruption of essential amino acid biosynthesis (lysine/tryptophan metabolism). These effects reinforce one another, ultimately producing a cascade of structural damage, energy depletion, and metabolic dysfunction. This multi-target mode of action may explain its strong activity against gastrointestinal pathogens and provides a mechanistic basis for the traditional use of Motuo citron in Tibetan medicine for digestive inflammatory disorders. From the perspective of practical application, plant-derived antibacterial phytochemicals can be loaded into porous chitosan-based aerogel carriers to improve their solubility, stability and antibacterial efficacy, offering a pathway to develop plant-based antibacterial materials for food safety applications [47].

Several limitations should be acknowledged in the present work. First, antibacterial evaluation was only performed against *S. aureus* and *E. coli,* so it remains unclear whether such inhibitory performance can be generalized to a broader spectrum of pathogenic bacteria. Second, the proposed multi-target antibacterial mechanism is inferred from phenotypic, metabolomic and biochemical assays of the complete essential oil, rather than being directly validated by dedicated mechanistic experiments. Third, although limonene, γ-terpinene and 3-carene were identified as predominant constituents, the current study did not clarify the individual antibacterial contribution of each compound, nor their potential synergistic or antagonistic interactions. Accordingly, mechanistic conclusions drawn in this work should be interpreted with caution. Further investigations using isolated single compounds and combinatorial mixing assays are required to dissect component-specific effects and verify the underlying molecular pathways.

## 5. Conclusions

Motuo *C. medica* essential oil, which is rich in monoterpenoids such as limonene, γ-terpinene, and 3-carene, showed strong antibacterial activity against *S. aureus* and *E. coli*, with greater activity against the Gram-positive strain. Mechanistic analyses showed that the essential oil disrupts bacterial cell wall and membrane integrity, inhibits key enzymes in glycolysis and the TCA cycle, and perturbs essential amino acid metabolism (lysine and tryptophan pathways). These coordinated effects suppress pathogenic bacterial growth through multiple interacting targets. This study provides mechanistic support for the ethnopharmacological use of Motuo citron in managing gastrointestinal infections and inflammatory conditions and provides a scientific basis for the broader utilization of Motuo *C. medica* resources in Xizang as a natural source of antimicrobial agents for pharmaceutical development.

## Figures and Tables

**Table 1:** Extraction yield and recovered mass of *C. medica* (Motuo citron) essential oil.

Group	Replicate	Pericarp Mass (g)	Recovered Oil Mass (g/100 g Pericarp) & Extraction Yield (%)
Enzyme-assisted steam distillation	1	100	0.47
	2	100	0.48
	3	100	0.49
	Mean ± SD	100	0.48 ± 0.01
Traditional steam distillation	1	100	0.15
	2	100	0.16
	3	100	0.17
	Mean ± SD	100	0.16 ± 0.01

**Table 2:** Relative contents of volatile compounds in citron essential oil.

Serial Number	Volatile Compound	Retention Time (min)	Relative Content (%)	Library Matching Score	CAS No.
1	Spiro[2,4]hepta-4,6-diene	4.826	0.16	75.93	765-46-8
2	2-methyl-5-(1-methylethyl)-Bicyclo[3.1.0]hex-2-ene	9.253	0.33	92.74	2867-05-2
3	(1R)-2,6,6-Trimethylbicyclo[3.1.1]hept-2-ene	9.456	1.01	95.16	7785-70-8
4	4-methylene-1-(1-methylethyl)-Bicyclo[3.1.0]hexane	10.699	0.17	89.83	3387-41-5
5	(1S)-6,6-dimethyl-2-methylene-Bicyclo[3.1.1]heptane	10.788	0.73	94.42	18172-67-3
6	.beta.-Myrcene	11.251	1.35	94.03	123-35-3
7	.alpha.-Phellandrene	11.649	0.46	91.10	99-83-2
8	3-Carene	11.836	5.22	96.67	13466-78-9
9	1-methyl-4-(1-methylethyl)-1,3-Cyclohexadiene	12.039	0.22	90.85	99-86-5
10	p-Cymene	12.275	3.04	93.20	99-87-6
11	D-Limonene	12.413	69.92	97.86	5989-27-5
12	trans-.beta.-Ocimene	12.705	0.27	90.15	3779-61-1
13	(1R)-2,6,6-Trimethylbicyclo[3.1.1]hept-2-ene	13.03	0.62	94.05	7785-70-8
14	gamma.-Terpinene	13.355	9.33	95.33	99-85-4
15	3-methyl-6-(1-methylethylidene)-Cyclohexene	14.208	0.09	81.07	586-63-0
16	1-methyl-4-(1-methylethylidene)-Cyclohexene	14.273	1.21	95.57	586-62-9
17	Linalool	14.598	0.17	79.21	78-70-6
18	2,6,6-Trimethyl-bicyclo[3.1.1]hept-3-ylamine	14.711	0.11	73.29	69460-11-3
19	(E,Z)-2,6-dimethyl-2,4,6-Octatriene	15.491	0.20	91.24	7216-56-0
20	6-Octenal, 3,7-dimethyl-, (S)-	16.186	0.11	79.07	5949-05-3
21	Terpinen-4-ol	16.937	0.08	76.90	562-74-3
22	.alpha.-Terpineol	17.319	0.17	80.65	98-55-5
23	2-Methyl-1,8-octanediol	17.717	0.08	78.66	1000433-27-9
24	(Z)-3,7-dimethyl-2,6-Octadien-1-ol	18.367	0.61	87.00	106-25-2
25	Neral	18.724	1.27	92.00	106-26-3
26	Citral	19.545	2.02	92.94	5392-40-5
27	2,6-Octadien-1-ol, 3,7-dimethyl-, acetate, (Z)-	22.071	0.26	88.86	141-12-8
28	2,6-Octadien-1-ol, 3,7-dimethyl-, formate, (Z)-	22.567	0.11	82.83	2142-94-1
29	Caryophyllene	23.663	0.22	82.66	87-44-5
30	(1R,5R)-2-Methyl-5-((R)-6-methylhept-5-en-2-yl)bicyclo[3.1.0]hex-2-ene	24.021	0.22	78.12	58319-06-5
31	Isobisabolene	25.8	0.21	89.04	1000424-85-5

**Table 3:** Effects of Motuo *C. medica* essential oil on bacteriostatic activity zone diameter.

Tested Bacteria	Diameter of Inhibition Zone (mm)		
	Citron Essential Oil	Positive Control	Blank Control
*S. aureus*	22.29 ± 0.82	25.37 ± 0.63	-
*E. coli*	11.85 ± 0.49	23.23 ± 0.58	-

**Table 4:** Effects of Motuo *C. medica* essential oil on MIC and MBC.

Tested Bacteria	Essential Oil Concentration (mg/mL)						Chloramphenicol	Negative Control	Blank Control
	1.625	3.75	7.5	15	30	60			
*S. aureus*	aA	aA	A	−	−	−	−	+	+
*E. coli*	bB	bB	bB	B	−	−	−	+	+

Note: “a and b” represent Minimum antibacterial concentration; “A and B” represent Minimum Bactericidal Concentration. “+” indicates colony growth; “−” indicates no colony growth.

## Data Availability

The original contributions presented in this study are included in the article. Further inquiries can be directed to the corresponding author.
